# Serological Screening Suggests Extensive Presence of* Mycoplasma gallisepticum* and* Mycoplasma synoviae* in Backyard Chickens in Southern Mozambique

**DOI:** 10.1155/2017/2743187

**Published:** 2017-01-22

**Authors:** Augusto Messa Júnior, Paula Taunde, Ana Felicidade Zandamela, Alberto Pondja Junior, Abel Chilundo, Rosa Costa, Custódio Gabriel Bila

**Affiliations:** ^1^Faculty of Veterinary Medicine, Eduardo Mondlane University, Av. de Moçambique Km 1,5, Maputo, Mozambique; ^2^Kyeema Foundation, Av. de Moçambique Km 1,5, Maputo, Mozambique

## Abstract

A total of 459 serum samples from unvaccinated backyard chickens originating from 4 villages in Mandlakazi district, Southern Mozambique, were tested for the presence of* Mycoplasma gallisepticum* and* Mycoplasma synoviae *antibodies through commercial enzyme-linked immunoabsorbent assay [ELISA] kits. Anti-MG and anti-MS antibodies were detected in all villages surveyed and the overall seroprevalence was 48.8% [95% CI 39.1–57.8] and 84.5% [95% CI 76.8–90.4], respectively. The risk of being seropositive for both diseases was higher [*P* < 0.05] in Chidenguele village than other villages. It is concluded that MG and MS serum antibodies are present in backyard chickens.

## 1. Introduction

Indigenous chickens are local breeds of chickens [*Gallus gallus domesticus*] reared in rural areas of most parts of the world. Commonly, no proper housing is provided and very little food supplementation is offered. They move freely, scavenging for food and water [1]. Nevertheless, these chickens provide eggs and meat to most rural and many urban consumers. In Mozambique, like many sub-Saharan African countries, the productivity of indigenous chicken is hampered by several infectious diseases. Moreover, it is widely believed that indigenous chickens may act as potential reservoirs for important poultry diseases [2].

Infectious disease such as avian mycoplasmosis is mentioned as a potential constraint to the health status and productivity of domestic chickens. The disease is mainly caused by two pathogens:* Mycoplasma gallisepticum* [MG] and* M. synoviae* [MS]. It causes considerable economic losses in chicken through reduction of weight gain and meat quality, increase in feed conversion rate in broilers, severe drop in egg production in layers, or increase in embryo mortality in breeders [3–6].

MG is bacteria belonging to the class Molliculates, family Mycoplasmataceae, and is the most important economically significant mycoplasma pathogen of poultry. MG infections are also known as chronic respiratory disease [CRD] of chickens, infectious sinusitis of turkeys, and house finch conjunctivitis [6, 7]. Birds of all age groups are more susceptible to this disease, but young birds are more prone to infection than adults [8]. MG may be transmitted horizontally from clinically infected or carrier birds and vertically through transovarian route [6, 9]. Chickens may have not obvious symptoms or may exhibit coughing, sticky nasal discharge, difficulty breathing, swelling of the face, sneezing, foamy secretion in the eyes, and a drop in body weight as well [9, 10].

MS is the etiological agent of acute to chronic respiratory disease in chickens. Similarly to MG, MS transmission is also horizontal or vertical. Although slight rales may be present in birds with* M. synoviae* respiratory infection, usually no clinical signs are noticed. When present, they include sneezing, nasal discharge, foam in the eye, rattle breathing, and swollen sinus. Morbidity is usually low to moderate with mortality of less than 10% [11, 12].

Literature on the epidemiology of avian mycoplasmosis in backyard chickens in Africa is scanty, with few reports in Zimbabwe [13], Botswana [14], Benin [15], South Africa [16], and Ethiopia [17]. To our knowledge, there are no published reports of this disease in either commercial or backyard chickens in Mozambique. The objective of this study was to assess the presence of avian mycoplasmosis in backyard chickens in Southern Mozambique using serological approach.

## 2. Materials and Methods

### 2.1. Study Area

**Figure 1:**
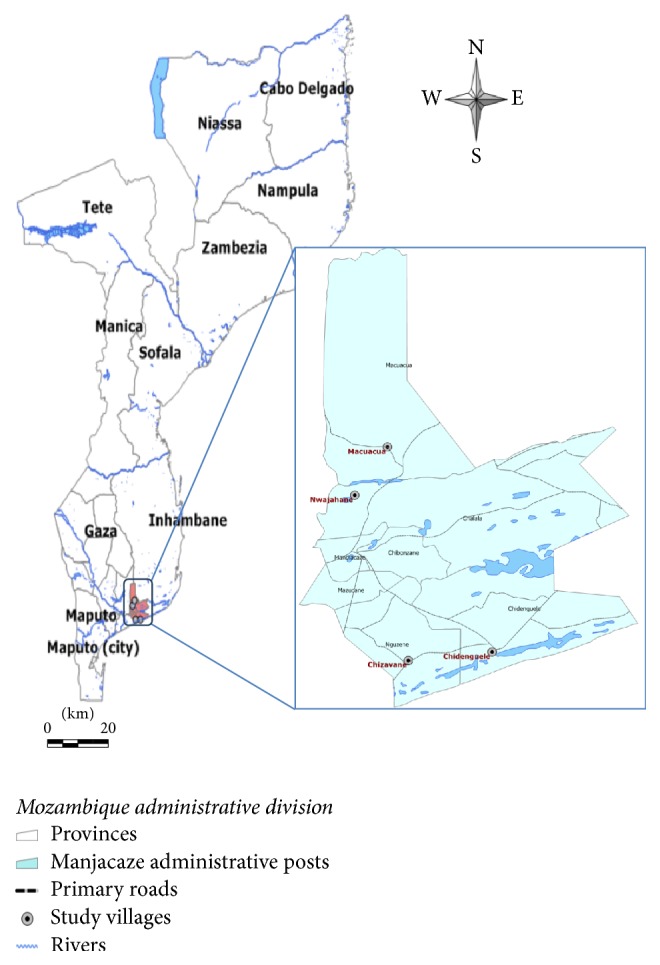
Map of Mozambique indicating the location of the study site.

The study was carried out in four villages in Mandlakazi district: Chidenguele, Macuacua, Chizavane, and Nwadjahane (Figure 1). Mandlakazi district is part of Gaza Province in Southern Mozambique and is located between latitudes 44.04°S and 25.00°S and longitudes 33.56°E and 34.28°E. It comprises an area of 3.797 km^2^ [18] with an estimated population of 175.607 inhabitants [19]. It is bordered in the north by Chibuto and Panda districts, in the south by Indian ocean, in the east by Zavala and Inharrime districts, and in the west by Chibuto and Xai-Xai.

The district is characterized by a dry inland and humid climate on the coast with rainfall varying from 400 mm to 950 mm per year, occurring from November to March, and with average monthly temperatures of 17 to 28°C [18]. It is administratively divided into seven areas (Chalala, Chibonzane, Chidenguele, Macuacua, Mazucane, Nguzene, and Manjacaze) and the mixed agriculture (livestock and crop) is the most important economic activity [18].

### 2.2. Study Design and Sampling

The study was conducted from January to March 2016, using a cross-sectional design. Willing and commitment with the objective of the study were the eligibility criteria for a villager to participate in the study. The sampling frame was all chicken keepers in the selected villages with chickens older than 2 months. Sample size calculation was done using the formula *n* = [*Zα*^2^*∗p∗q*/*L*^2^], where *n* = sample size required; *Zα* = 1.96 is the value required for confidence of 95%; *p* is the a priori estimate of the prevalence; *q* = 1 − *p* , the complementary of prior estimate, and *L* = 5%, the precision of estimate, given by Naing et al. [20]. A priori estimate of the prevalence of 50% was used, once there were no previous studies regarding avian mycoplasmosis. A sample size of 459 was computed.

### 2.3. Blood Sampling and Serology

Approximately 2.5–3 mL of blood was collected from brachial vein per each chicken into disposable syringes, as described by Kelly and Alworth [21]. Then, the syringes were left horizontally and then vertically for the serum to ooze out. Serum was then collected in 2 mL cryovial tubes and kept at −20°C until testing.

Serum samples were analysed using commercial ELISA kits for the presence of anti-MG antibodies [ProFLOK®*Mycoplasma gallisepticum* Antibody Test Kit, Synbiotics Corp., San Diego, CA, item number 96-6533] and anti-MS antibodies [ProFLOK®*Mycoplasma synovie* Antibody Test Kit, Synbiotics Corp., San Diego, CA, item number 96-6536], according to the manufacturer's instructions. These commercial kits are based on the principle of indirect ELISA. The sample and control OD values were read using an automated microplate reader [EL ×800, BIOTEK, Instruments Inc., Winooski, VT] at 405 nm. For each sample, the sample-to-positive [*S*/*P*] ratios were calculated from OD values by the formula:

(1) S/P ratio=OD sample−negative control mean ODpositive control mean OD−negative control mean OD;

see [22].

### 2.4. Data Analysis

Data were entered in MS Excel spreadsheet and exported to STATA® version 12.1 [Stata IC 12.1 for Windows], software for analysis. Prevalence data were analysed using chi-square test [*χ*^2^-test]. Logistic regression models were used to compute odds ratios [OR] to identify the risk for being seropositive as dichotomous dependent variable and independent variable [location]. In all chi-square tests, a probability level of *P* < 0.05 was considered statistically significant.

## 3. Results and Discussion

The backyard chickens tested in this study had no previous history of avian mycoplasmosis vaccination since this practice is not routine in the poultry industry in Mozambique. Hence, the presence of antibodies to MG and MS in all surveyed villages was considered clear evidence that the birds have been naturally exposed to those two infectious agents.

The MG prevalence observed in this study was 48.8%, ranging from 28.5% to 72.4% (Table 1). Our finding constitutes the first report of MG prevalence in poultry in Mozambique and is approximately in agreement with report from Zimbabwe (<33%) [13]. Higher prevalence was reported in backyard chickens in Benin (62%) [15], Botswana (57.8%) [14], South Africa (63%) [16], Ecuador (73%) [22], Bangladesh (58.9%) [23], Argentina (68.6–100%) [24], Ethiopia (67.7%) [17], and Brazil (53.3%) [25].

Our study also revealed that the prevalence of MS antibodies was generally high, around 84.5%, and varied from 68.2% to 95.8% (Table 1). Our results are the first documentation of the presence of MS in backyard chickens in Mozambique and are consistent with seroprevalence in fancy breeding chickens of 75% in Switzerland [26], where the management system is equivalent to the one in backyard poultry flocks. Lower prevalence was reported in Botswana (40.99%) [14] and Paraguay (53%) [27].

In the studied villages, the backyard chickens were neither given any immunizations nor afforded treatments, which make them intrinsically sensitive to numerous infectious diseases. These chickens were on poor plane of nutrition and were kept in flocks of mixed ages, with susceptible chicks in contact with adults that are potential reservoirs for diseases. Furthermore, they were allowed to move freely, scavenging for food and water in the crop fields, lakes, and rivers, habitats that attract large numbers of wild birds. These epidemiology factors may have contributed to the natural exposure of the birds and could explain the high prevalence detected for both MG and MS, as reported elsewhere [28].

In terms of geographical variations, the seroprevalence of both MG and MS was higher in Chidenguele than other three villages with 63.9% and 91.8%, respectively (Table 1). Why the seroprevalence is higher in this village compared to other villages is unknown. However, it could be related to the proximity of Chidenguele village to the main national road [N1], where there is huge influx of people and animals moving from south to north regions of the country and vice versa.

## 4. Conclusions

There was a serological evidence of the presence of MG and MS in backyard chickens in Mandlakazi district of Mozambique.

## Figures and Tables

**Table 1:** Seroprevalence of MG and MS in unvaccinated backyard chickens in Southern Mozambique.

Villages	Number of serum samples	Seroprevalence [%, exact 95% CI]	
		MG	MS
Nwadjahane	103	37.8 [28.5–47.9]^a^	89.3 [81.7–94.5]^a^
Macuacua	106	48.1 [38.3–58.0]^a^	77.3 [68.2–84.9]^b^
Chizavane	128	43.8 [35.0–52.8]^a^	79.6 [71.7–86.3]^b^
Chidenguele	122	63.9 [54.7–72.4]^b^	91.8 [85.4–95.8]^a^
*Total*	*459*	*48.8 [39.1–57.8]*	*84.5 [76.8–90.4]*

^a,b^Villages with different superscript differ significantly (*P* < 0.05).
